# Silymarin Synergizes with Antiviral Therapy in Hepatitis B Virus-Related Liver Cirrhosis: A Propensity Score Matching Multi-Institutional Study

**DOI:** 10.3390/ijms25063088

**Published:** 2024-03-07

**Authors:** Chien-Hao Huang, Victor Chien-Chia Wu, Chun-Li Wang, Chia-Ling Wu, Yu-Tung Huang, Shang-Hung Chang

**Affiliations:** 1 Division of Hepatology, Department of Gastroenterology and Hepatology, Chang Gung Memorial Hospital, Linkou Medical Center, Taoyuan City 33305, Taiwan; q12248@cgmh.org.tw; 2 School of Medicine, College of Medicine, Chang Gung University, Taoyuan City 33305, Taiwanwang3015@cgmh.org.tw (C.-L.W.); 3 Department of Cardiology, Chang-Gung Memorial Hospital, Linkou Medical Center, Taoyuan City 33305, Taiwan; 4 Center for Big Data Analytics and Statistics, Department of Medical Research and Development, Chang Gung Memorial Hospital, Linkou Medical Center, Taoyuan City 33305, Taiwan; dorycwu@cgmh.org.tw (C.-L.W.);

**Keywords:** silymarin, antiviral therapy, hepatitis B virus, liver cirrhosis, mortality, HCC occurrence, synergic effect

## Abstract

Hepatitis B virus (HBV)-related liver cirrhosis (HBV-LC) presents a substantial mortality and hepatocellular carcinoma (HCC) risk. While antiviral therapy (AVT) is the standard, complete HBV clearance remains elusive and may not reduce the risk of death in patients with decompensated cirrhosis. Silymarin, a centuries-old herbal remedy, has shown promise against HBV infection and as an antifibrosis therapy. This study explores the potential of silymarin combined with AVT to reduce mortality and HCC incidence in patients with HBV-LC. This research, spanning from 2001 to 2019, entailed a multi-institutional retrospective cohort study which included 8447 HBV-LC patients all undergoing AVT. After applying inclusion and exclusion criteria, the study comprised two cohorts: a case cohort receiving silymarin alongside AVT for at least 30 days, and a control cohort on AVT alone. Propensity score matching, based on baseline parameters including HBV-DNA levels, comorbidity, and an important LC medication, namely, non-selective β-blockers, was employed to ensure balanced groups, resulting in 319 patients in each cohort for subsequent analyses. Overall mortality was the primary outcome, with HCC occurrence as a secondary outcome. Among 319 patients in both cohorts, the case cohort exhibited significant improvements in the international normalized ratio (INR), model for end-stage liver disease (MELD) score and the Charlson comorbidity index (CCI) one year after the index date. A competing risk survival analysis demonstrated superior one-year and two-year mortality outcomes in the case cohort. However, no significant impact on one-year and two-year HCC occurrence was observed in either cohort. The combination of silymarin and AVT in HBV-LC patients demonstrated a synergistic effect, leading to decreased overall mortality and an improved comorbidity index. While the incidence of HCC remained unchanged, our results suggested promising potential for further clinical trials investigating the synergistic role of silymarin in the treatment of HBV-LC.

## 1. Introduction

Hepatitis B virus (HBV) infection poses a significant global health burden, leading to hepatic decompensation, liver cirrhosis (LC), and hepatocellular carcinoma (HCC) [1,2]. With approximately 290 million people infected worldwide, the Asia Pacific region is heavily affected, with an annual death toll of around 1 million [3,4]. In Taiwan, HBV prevalence was once as high as 15–20% before the implementation of the viral hepatitis control program (VHCP) in 1984 [5]. The progression of chronic HBV infection to chronic hepatitis occurs in a substantial proportion of patients, with 20–25% experiencing repeated hepatitis flares annually and 3–4% per year developing LC, necessitating consideration for antiviral therapy (AVT) [1,4].

LC is an advanced stage of chronic liver disease characterized by extensive fibrosis and disruption of normal liver architecture [6]. Studies have demonstrated the significant clinical benefits of AVTs such as lamivudine in delaying disease progression and reducing the risk of hepatic decompensation and HCC in patients with chronic hepatitis B and advanced fibrosis or cirrhosis [7]. High genetic barrier nucleoside/nucleotide analogues (NAs), like entecavir (ETV), have also shown remarkable efficacy in reducing the risks of hepatic events, HCC, and liver-related and all-cause mortalities in patients with HBV-related liver cirrhosis (HBV-LC) over a 5-year period, particularly among those who maintained viral suppression [8]. For patients with decompensated cirrhosis, the early initiation of AVT, irrespective of HBV DNA level, is recommended, along with standard care and liver transplantation (LT) evaluation [9,10]. However, it should be noted that these AVT options are not able to clear covalently closed circular DNA (cccDNA), the replication template for HBV in hepatocyte nuclei [11,12,13]. Despite AVT treatment, a proportion of compensated cirrhotic patients (3.9%) still progress to hepatic decompensation [14,15], leading to a poor prognosis [9,16]. While AVT significantly alters the natural course of decompensated cirrhosis, improving liver function and increasing survival, a subset of patients (13.4–16.2%), particularly those with severe hepatic dysfunction, face mortality within 6 months [17,18]. A recent study has even shown that AVT may not lower the risk of death in patients with decompensated cirrhosis [19]. These results highlight the importance of promptly administering potent AVT to patients under consideration for LT [17] and also underscore the need for complementary treatment strategies alongside AVT alone.

Silymarin, derived from the milk thistle herb (silybum marianum), is commonly used by patients with chronic viral hepatitis to decrease transaminase level, but its efficacy remains largely unknown [20]. A systemic review commented that silymarin and its principal phytoconstituent, silibinin, play an important role in the prevention and treatment of HCC [21]. An in vitro study demonstrated that silibinin, a major extract of silymarin, inhibits HBV entry into hepatocytes via the inhibition of clathrin-mediated endocytosis (CME) [11]. In addition, the combination of silibinin and ETV reduced HBV DNA levels in HepG2-NTCP-C4 cells that had already been infected with HBV, suggesting a possible synergic effect in combination therapy in patients with chronic HBV infection [11].

Moreover, the molecular mechanisms underlying LC involve chronic liver injury, inflammation, and the activation of hepatic stellate cells (HSCs) [22]. Persistent liver inflammation leads to the release of pro-inflammatory cytokines and chemokines. Silymarin, studied for its potential therapeutic effects on LC, is known to possess antioxidant properties. It scavenges free radicals and reduces oxidative stress implicated in the progression of liver fibrosis [23]. Additionally, it exhibits anti-inflammatory effects by modulating various inflammatory pathways. Furthermore, silymarin has been reported to interfere with the activation of HSCs [24], potentially inhibiting the key cellular process responsible for fibrosis. The hepatoprotective effects of silymarin involve a combination of antioxidative, anti-inflammatory, and antifibrotic mechanisms [25], making it a subject of interest in the management of LC.

Considering the increased risk of death and HCC in patients with hepatic flares and HBV-LC [26,27], coupled with our limited knowledge regarding the efficacy of combination therapy with anti-HBV agents and silymarin in LC (while safety is noted, the meta-analysis does not draw a clear conclusion on the efficacy of silymarin for treating chronic hepatitis B) [28], the aim of this study is to investigate whether adding silymarin to anti-HBV treatment can reduce mortality and the incidence of HCC in patients with HBV-LC.

## 2. Results

### 2.1. Flowchart

**Figure 1:**
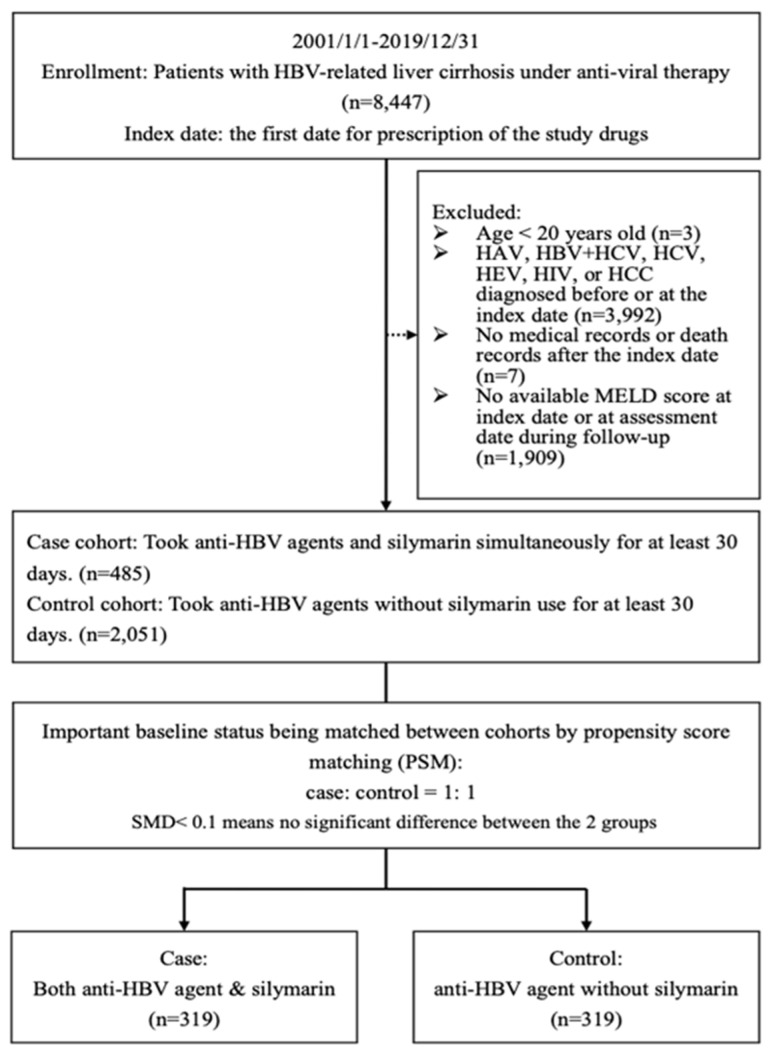
Flowchart.

Between 1 January 2001 and 31 December 2019, a total of 8447 patients with HBV-LC, who received AVT according to the Asian Pacific Association for the Study of the Liver (APASL) [29,30,31,32] and the Taiwan national health insurance [33,34] guidelines, were initially considered for eligibility from the electronic medical records of the multi-institutional Chang Gung Memorial Hospital system (Figure 1). Patients under 20 years old; those with previous coinfection or superinfection of hepatitis A virus (HAV), coinfection or superinfection of hepatitis B and C virus (HBV + HCV), hepatitis C virus (HCV), hepatitis E virus (HEV), or human immunodeficiency virus (HIV); those diagnosed with HCC before or at the index date; and those with incomplete relevant follow-up records were excluded. Finally, a cohort of 2536 patients were enrolled in this study. These patients were then divided into two cohorts: a case cohort (*n* = 485) for those who received simultaneous AVT and silymarin treatment for at least 30 days and a control cohort (*n* = 2051) for those who received AVT alone for at least 30 days. To ensure comparability between the two cohorts, propensity score matching (PSM) was conducted to match important baseline factors, including demographic data, creatinine (Cr), sodium (Na), aspartate aminotransferase (AST), alanine aminotransferase (ALT), albumin, HBV-DNA, international normalized ratio (INR), decompensation status, model for end-stage liver disease (MELD) score, albumin–bilirubin (ALBI) score, Charlson comorbidity index (CCI), and cumulative duration of medication. After PSM, 319 patients from both the case and control cohorts were selected for further analysis.

### 2.2. Clinical Characteristics in the Two Propensity Score-Matched Cohorts

Table 1 presents the comparison of baseline clinical characteristics between PSM case and control cohorts of patients with HBV-LC who received AVT with and without silymarin (matched at a ratio of 1:1). Males were predominant in both the case cohort and control cohorts. Following propensity score matching, there were no significant differences in any of the listed parameters, including age, sex, Cr, Na, AST, ALT, albumin, HBV-DNA levels, INR, baseline decompensation status (esophageal varices (EV)/gastric varices (GV) bleeding, ascites, hepatic encephalopathy, and hepatorenal syndrome), clinical index (MELD score and ALBI score), CCI, and cumulative duration of medication (ASMD < 0.1 indicates no significant difference between the two groups).

### 2.3. Comparison of the Cumulative Duration of Study Medication, Follow-Up Time, and Primary and Secondary Outcomes of the Two Cohorts

Table 2 shows the cumulative duration of study medication, other important drugs for liver cirrhosis, like the non-selective β-blocker (NSBB), follow-up time, and primary and secondary outcomes of the two cohorts. The cumulative duration of study medication was similar between the case and control cohorts. There was no significant difference in the usage of NSBB between the two groups. The mean follow-up period was significantly longer in the case cohort than in the control cohort. Regarding the primary outcome, there was a significantly higher mortality rate in the control cohort (59.87%) compared to the case cohort (48.28%), as well as a difference in the liver transplantation (LT) rate. As for the secondary outcome, there were significant differences in HCC incidence and rates of cirrhotic complications, including EV/GV bleeding, ascites, hepatic encephalopathy, and hepatorenal syndrome.

### 2.4. Comparing Laboratory Parameters and Clinical Indices of the Two Cohorts

We then examined whether there were improvements in laboratory values and clinical indices one year after the index date in both the case and control cohorts. As presented in Table 3, there were no significant differences in the improvement of serum Cr, Na, AST, ALT, albumin, HBV-DNA, or the ALBI score between the two cohorts of patients with HBV-LC. However, there was a significant difference in the improvement of INR, MELD score, and CCI in the case cohort compared to the control cohort for patients with HBV-LC. The changes in laboratory parameters and clinical indices one year after the index date in the individual cohort of patients with HBV-LC are presented in Supplementary Table S1. Notably, significant improvements were observed in most of the laboratory parameters and clinical indices, both in the case cohort and the control cohort.

### 2.5. Competing Risk Analysis for the Primary Outcome

**Figure 2:**
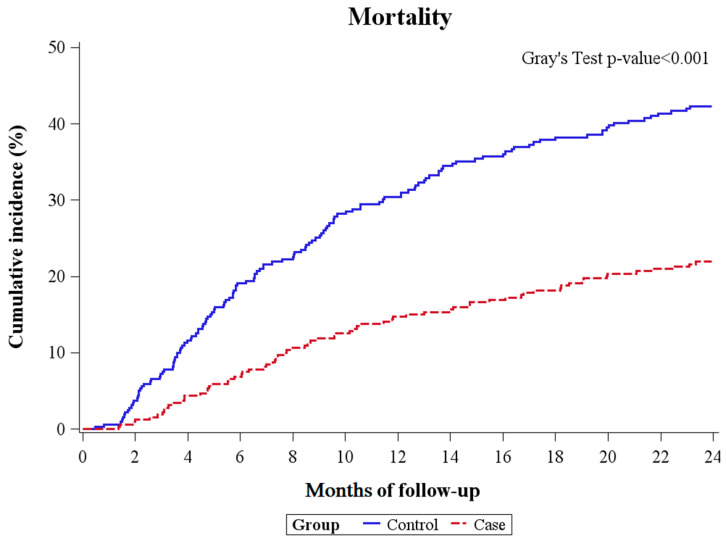
Mortality over one year and two years.

A competing risk analysis was performed to analyze whether the case or control cohort could independently predict overall follow-up mortality, considering LT as the competing risk. As shown in Table 4 and Figure 2, the case cohort showed a significantly lower hazard ratio (HR) for both one-year and two-year mortality.

Since mortality is affected by medical advancement, i.e., a patient who has the same medical condition would have a much higher chance of surviving in 2019 than in 2003, an additional analysis was conducted to explore the distribution of patient index dates across different years. While there was a statistically significant variance in the patient index year distribution (Supplementary Table S2, ASMD > 0.1 indicating statistical significance), incorporating the index year variable into our original competing risks Cox regression model did not alter the outcome of the analysis (Supplementary Tables S3 and S4). The consistency of these results with the original Table 4 and Table 5 supports the robustness of our findings, irrespective of the distribution differences in patient index years. 

### 2.6. Competing Risk Analysis for the Secondary Outcome

**Figure 3:**
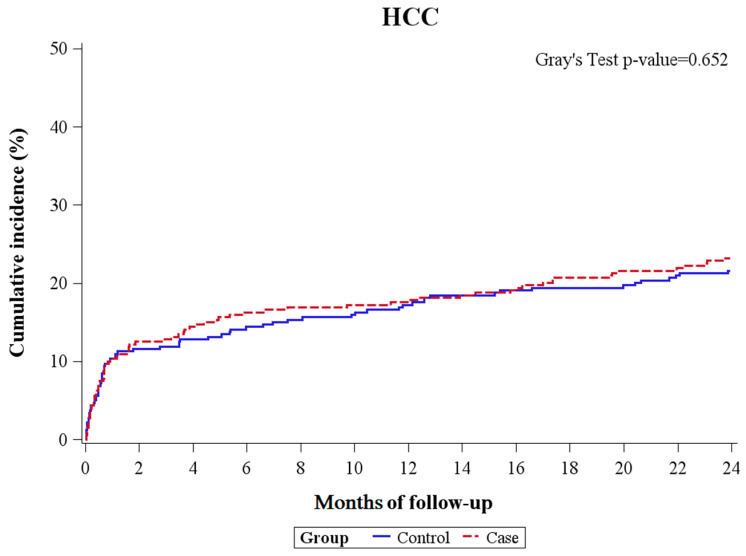
First HCC occurrence over one year and two years.

A competing risk analysis was conducted to investigate whether the case or control cohort could independently predict the occurrence of HCC, considering mortality or LT as the competing risks. Table 5 and Figure 3 collectively demonstrate that cohort status did not exert a significant impact on the HR for either one-year or two-year HCC occurrence.

## 3. Discussion

In this multi-institutional retrospective study utilizing propensity score matching, we aimed to investigate whether the combination of silymarin and anti-HBV agents could synergistically reduce mortality and/or the incidence of HCC in patients with HBV-LC. Patient data meeting inclusion and exclusion criteria were extracted from the electronic medical records of the Chang Gung Memorial Hospital system and categorized into either a case cohort, receiving simultaneous silymarin and AVT treatment for at least 30 days, or a control cohort, receiving AVT alone for the same duration. Following PSM to align key baseline parameters including demographics, AST, ALT, HBV-DNA, decompensation status, MELD score, ALBI score, CCI, and cumulative duration of medication, 319 patients were analyzed in each cohort. After one and two years from the index date, notable improvements were observed in INR, MELD score, and CCI within the case cohort compared to the control cohort among patients with HBV-LC. A competing risk analysis was employed to independently evaluate the predictive capacity of the case and control cohorts for overall follow-up mortality, considering LT as a competing risk. The analysis revealed a significantly lower hazard ratio (HR) for mortality in the case cohort at both one-year and two-year follow-ups. Similarly, a competing risk analysis was conducted to assess the cohorts’ independent predictive ability for HCC occurrence, accounting for mortality or LT as competing risks. The outcome indicated that cohort status did not exert a significant influence on the HR for either one-year or two-year HCC occurrence. While the combination of silymarin and AVT did not exhibit a substantial reduction in HCC incidence when compared to AVT alone, our findings suggest a potential beneficial impact on overall mortality. These results provide promising insights for future clinical trials aimed at exploring the synergic therapeutic role of silymarin in the treatment of HBV-LC.

Investigating the effects of silymarin in combination with standard antiviral treatment is of value due to its widespread use by patients with chronic viral hepatitis [20,35]. A previous comprehensive review indicated that while milk thistle treatment was safe and well-tolerated, it did not lead to reduced mortality, improved liver function markers, or histological enhancements in chronic liver disease patients [36]. However, the result on mortality was based on the result of only four trials including 433 subjects, and it included all etiologies of liver disease [36]. A subsequent systemic review argued that silymarin combined with antiviral drugs significantly reduced the level of serum transaminases, hepatic fibrosis markers, and serum transforming growth factor (TGF)-β1, tumor necrosis factor (TNF)-α, and interleukin (IL)-6 versus antiviral drugs in patients with CHB [28]. Nevertheless, this review concluded that there was insufficient evidence to recommend the combination of silymarin and antiviral drugs for CHB treatment [28]. Our findings, indicating an additional benefit in overall mortality along with reductions in serum transaminases, hepatic fibrosis scores, HBV-DNA, and CCI through the addition of silymarin to standard AVT, align with and reinforce the observations made in the mentioned review.

Blessed milk thistle, also known as silybum marianum, is a flowering plant indigenous to Mediterranean Europe [21]. It has been consumed and widely used to treat various chronic liver diseases over the centuries [37]. Silymarin, the extract from milk thistle seed, is particularly recognized for its potent antioxidant and anti-inflammatory properties, contributing to its therapeutic and hepatoprotective effects [21]. Silibinin, the principal phytoconstituent of silymarin, showcases an array of attributes such as antioxidant, immunomodulatory, antiproliferative, antifibrotic, and anticancer activities [38,39,40], spanning a wide range of tissues and organs [41,42]. Notably, an in vitro study demonstrated silibinin’s ability to impede HBV entry into hepatocytes by inhibiting CME [11]. Furthermore, both silymarin and silibinin exhibit varying degrees of hindrance against HCV infection in cell culture, acting on viral entry, fusion, RNA and protein synthesis, HCV NS5B RNA-dependent RNA polymerase activity, and virus transmission [41,43,44,45]. Clinically, silymarin has been shown to inhibit HCV and, to a lesser extent, HIV-1, in one HCV/HIV coinfected case [46], and even as an adjuvant therapy to enhance viral eradication rate prior to the direct-acting antiviral (DAA) era [47]. However, this phenomenon lacks validation in the context of patients with CHB.

Few studies have explored the combined use of silymarin with interferon, nucleoside/nucleotide analogues, or other conventional treatments, specifically in cirrhotic patients with pure CHB [20,28], excluding those with HCC [48]. Therefore, the value of this study lies in verifying the synergistic role of silymarin combined with AVT, leading to reduced mortality while not preventing early HCC occurrence in a susceptible HBV-LC patient cohort. A randomized controlled trial (RCT) has demonstrated silymarin’s association with a noteworthy decrease in liver-related deaths among cirrhosis patients [37,49]. Compelling evidence also underscores that AVT alone substantially lowers the risk of death and HCC compared to placebos in patients with HBV-LC or HBV-ACLF [50,51]. These anti-HBV agents primarily function by robustly suppressing HBV-DNA, which is associated with disease progression risk [52] and HCC development [53]. Moreover, silymarin may potentially enhance AVT by exhibiting antifibrotic effects [47], mitigating oxidative stress induced by ROS, and suppressing sustained hepatic inflammation through modulation of the prostaglandin pathway in animal models [54]. Therefore, it is reasonable to assume that silymarin enhances AVT’s impact on reducing overall mortality. However, achieving a significant reduction in the primary prevention of HCC incidence through silymarin in combination with AVT, compared to AVT alone, might be challenging. This is because the annual incidence of HCC occurrence in HBV-LC is much lower than the mortality incidence [55,56]. Furthermore, current findings suggest that both conjugated and unconjugated silymarin undergo rapid elimination in vivo [57]. In contrast, nanoformulated silymarin demonstrates an extended-release profile at the administration site, potentially reducing the risk of adverse effects [57] and providing enhanced efficacy against HCC. Therefore, meticulous clinical trial design and execution are crucial to ascertain these advantages before nanoformulated silymarin can be considered for market release.

The hepatoprotective effects of silymarin involve various molecular mechanisms, with a focus on the Nrf2 (nuclear factor erythroid 2-related factor 2) pathway [58]. The Nrf2 pathway functions as a cellular defense mechanism, crucial for protecting cells from oxidative stress and inflammation. Studies suggest that silymarin may activate the Nrf2 pathway, serving as a negative regulator of chronic inflammation in LC [59]. Additionally, considering the central role of HSCs in liver fibrosis [60], silymarin has been proposed to inhibit their activation [24]. By intervening at this cellular level in the fibrotic process, silymarin holds the potential to prevent or reduce the progression of LC.

Our study has certain limitations, with the primary one being its retrospective nature. Nonetheless, the study involved a substantial cohort of 2536 HBV-LC patients sourced from the multi-institutional Chang Gung Memorial Hospital system. Propensity score matching was meticulously employed to align crucial baseline factors such as demographics, Cr, Na, AST, ALT, albumin, HBV-DNA, INR, decompensation status, MELD score, ALBI score, comorbidity index (CCI), and cumulative duration of medication. Furthermore, competing risk analysis was conducted, accounting for liver transplantation as a potential confounding factor. Another limitation is the exclusion of etiologies other than HBV, which restricts the generalization of our findings to conditions like HCV or NAFLD, where silymarin’s effects may have a stronger evidence base [61].

## 4. Materials and Methods

### 4.1. Study Design and Data Source

A multi-institutional retrospective study was conducted within the Chang Gung Memorial Hospital (CGMH) system, which is the largest healthcare system in Taiwan, comprising three medical centers (Taipei CGMH, Linkou CGMH, and Kaohsiung CGMH), three regional hospitals (Keelung CGMH, Taoyuan CGMH, and Chiayi CGMH), and one district hospital (Yunlin CGMH) located from the northeast to southern regions of Taiwan [62,63]. Data were retrieved from the electronic medical records (EMRs) containing outpatient, emergency, and inpatient claim records, as well as laboratory, drug, imaging, endoscopy, and microbiology reports. More detailed information has been reported in a previous study [64]. The study protocol conforms to the ethical guidelines of the 1997 Declaration of Helsinki and was approved by the ethical committee of the Chang Gung Memorial Hospital (202000112B0).

### 4.2. Study Population

As delineated in the enrollment flowchart (Figure 1), the inclusion criteria encompassed all consecutive adult patients diagnosed with HBV-LC who were taking AVT according to the APASL [29,30,31,32] and Taiwan National Health Insurance [33,34] guidelines recorded in the Taiwanese Chang Gung Research Database EMR between 1 January 2001 and 31 December 2019. The exclusion criteria encompassed individuals under the age of 20; those with previous coinfection or superinfection of HAV, HBV + HCV, HCV, HEV, or HIV, or those diagnosed with HCC before or at the index date; and those with incomplete relevant follow-up records, such as MELD scores, at the index date or during subsequent assessment dates. Patients were assigned to one of two cohorts. The case cohort consisted of patients who received simultaneous AVT and silymarin treatment for a minimum of 30 days, while the control cohort encompassed patients who received AVT alone without silymarin usage for a minimum of 30 days [65]. The daily dose of silymarin was 150 mg administered twice to three times a day [65].

### 4.3. Diagnostic Criteria for HBV-Related Liver Cirrhosis with AVT

The diagnosis of liver cirrhosis was primary established using the International Classification of Diseases, 9th Revision, Clinical Modification (ICD-9-CM) diagnosis code (571.2, 571.5, 571.6) or the 10th Revision (ICD-10) code K70.3, K74.3, K74.5, K74.6, K71.7 in conjunction with confirmation by abdominal echography. Notably, cases of alcoholic liver cirrhosis (i.e., 571.2; K70.3) were excluded.

Furthermore, the diagnosis of HBV-related liver cirrhosis under AVT was established based on the aforementioned criteria plus laboratory examinations indicating HBsAg or HBsAg quantification or HBV-DNA positivity. Additionally, individuals were required to have received at least 30 days of treatment with the listed NA (lamivudine, adefovir, entecavir, telbivudine, tenofovir, vemlidy) or interferon (peginterferon alpha-2a, interferon alpha-2a) therapies, as confirmed by National Health Reimbursement Insurance (NHRI) Hepatitis B Carriers and Hepatitis C Infected Patient Medical Benefit Improvement Program (P4201C or P4202C) payment records.

### 4.4. Index Date and Follow-Up Time

The index date was defined as the earliest date of the administration of the prescribed study drug. The follow-up time was defined as the duration between the index date and either the occurrence of mortality or the latest medical record within the study period.

### 4.5. Calculation of Medication Duration

The case group primarily consisted of individuals with a cumulative duration of concomitant anti-HBV and silymarin medication use, while the control group focused on the cumulative duration of anti-HBV medication. After propensity score matching, the cumulative duration of medication days was matched between the study groups. Days during which treatment was interrupted (defined as a lapse of three months or more without medication) were excluded from the calculation.

### 4.6. Propensity Score Matching

To ensure comparability between the two cohorts, propensity score matching (PSM) was conducted to match important baseline factors, including demographic statistics, Cr, Na, AST, ALT, albumin, HBV-DNA, INR, decompensation status, MELD score, ALBI score, comorbidity index (CCI), and cumulative duration of medication using a 1:1 PSM.

### 4.7. Primary and Secondary Outcomes

The primary outcome was defined as overall mortality within one-year and two-year follow-up periods, with liver transplantation (LT) considered as a competing risk event. The secondary outcome was defined as the occurrence of HCC within the one-year and two-year follow-up period, with mortality or LT considered as competing risk events.

### 4.8. Asessing the Magnitude of Change in Laboratory Parameters, Clinical Index, and Charlson Comorbidity Index

The magnitude of change was determined by subtracting the data at the index date from the data recorded one year after the index date.

### 4.9. Statistical Methods

Descriptive statistics were employed to present continuous variables as mean ± standard deviation (SD) and categorical variables as frequencies and percentages. Important baseline factors, such as age, sex, Cr, Na, AST, ALT, albumin, HBV-DNA, baseline decompensation status (EV/GV bleeding, ascites, hepatic encephalopathy, and hepatorenal syndrome), clinical index (MELD score and ALBI score), and Charlson comorbidity index (CCI), were subjected to 1:1 PSM to achieve comparability between the case and control cohorts. An absolute standardized mean difference (ASMD) < 0.1 indicated no significant difference between the groups post PSM. The comparison of the magnitude of change in Table 3 was conducted using an independent *t*-test, while changes in laboratory parameters and clinical indicators one year after the index date were assessed using a paired-sample *t*-test.

Competing risks analyses were performed to assess the hazard ratio of the primary and secondary outcomes over a span of 1 and 2 years, respectively. A *p* value of <0.05 was considered statistically significant. All statistical analyses were performed using SAS software, version 9.4 (SAS Institute, Cary, NC, USA).

## 5. Conclusions

The combined use of silymarin and AVT in HBV-LC patients demonstrated a synergistic effect, resulting in reduced overall mortality and improvements in INR, MELD score, and the Charlson comorbidity index. While the incidence of HCC was not significantly impacted, our results suggest a promising avenue for future clinical trials investigating silymarin’s synergic role in HBV-LC treatment. Further exploration of silymarin’s role in HBV-related non-cirrhotic patients also warrants additional investigation.

## Figures and Tables

**Table 1:** Comparison of baseline clinical characteristics between propensity score-matched CHB cirrhotic patient cohorts who took antiviral agents with silymarin use vs. without silymarin use (1:1).

Baseline Clinical Characteristics @	Case (Anti-HBV + Silymarin) (*n* = 319)	Control (Anti-HBV Agent) (*n* = 319)	ASMD ^#^
Age (years), mean ± SD	55.94 ± 11.47	55.93 ± 13.03	0.001
Sex, n (%)			0.022
Male	241 (75.55)	244 (76.49)	
Female	78 (24.45)	75 (23.51)	
Baseline biochemistry			
Creatinine (mg/dL)	1.16 ± 1.31	1.09 ± 1.39	0.050
Na (mEq/L)	137.48 ± 4.11	137.43 ± 4.31	0.014
AST (U/L)	122.69 ± 205.76	123.27 ± 246.9	0.003
ALT (U/L)	114.7 ± 231.02	106.41 ± 214.87	0.037
Albumin (g/dL)	3.27 ± 0.75	3.22 ± 0.73	0.079
HBV-DNA (log_10_ IU/mL)	7.37 ± 8.01	7.47 ± 8.1	0.053
Hemogram			
INR	1.27 ± 0.33	1.26 ± 0.43	0.012
Baseline decompensation status, n (%)			
EV or GV bleeding	75 (23.51)	81 (25.39)	0.044
Ascites	127 (39.81)	115 (36.05)	0.078
Hepatic encephalopathy	27 (8.46)	35 (10.97)	0.085
Hepatorenal syndrome	1 (0.31)	1 (0.31)	0.000
Clinical index			
MELD score	12.89 ± 5.47	12.91 ± 5.7	0.003
ALBI score	−1.77 ± 0.77	−1.71 ± 0.76	0.081
CCI (Charlson comorbidity index), mean ± SD	1.93 ± 2.43	2.11 ± 2.63	0.072

@: values are expressed in n (%) or mean ± standard deviation; case: silymarin + anti-HBV agents (IFN or nucleoside/nucleotide analogs); control: anti-HBV agents alone; ASMD **^#^**: absolute standardized mean difference. SMD < 0.1 means no significant difference between the two groups; EV: esophageal varices; GV: gastric varices.

**Table 2:** Follow-up time, primary and secondary outcomes of the two cohorts of patients with HBV-related liver cirrhosis.

	Case (Anti-HBV + Silymarin) (*n* = 319)	Control (Anti-HBV Agent) (*n* = 319)	ASMD ^#^
Cumulative duration of medication, mean ± SD (months)	9.11 ± 13.33	9.62 ± 12.95	0.039
Non-selective β-blocker	31 (9.72)	24 (7.52)	0.078
Follow-up time, mean ± SD (months)	61.97 ± 47.77	42.24 ± 49.24	0.407
Primary outcome, n (%)			0.349
Mortality	154 (48.28)	191 (59.87)	
LT ^$^	6 (1.88)	18 (5.64)	
Secondary outcome, n (%)			
HCC	107 (33.54)	91 (28.53)	0.109
Cirrhotic complications, n (%)			
EV or GV bleeding	62 (19.44)	86 (26.96)	0.179
Ascites	23 (7.21)	33 (10.34)	0.111
Hepatic encephalopathy	6 (1.88)	14 (4.39)	0.144
Hepatorenal syndrome	107 (33.54)	91 (28.53)	0.109

Case: silymarin + anti-HBV agents (IFN or nucleoside/nucleotide analogs); control: anti-HBV agents; ASMD **^#^**: absolute standardized mean difference. SMD < 0.1 means no significant difference between the two groups; LT ^$^: liver transplantation.

**Table 3:** Comparisons of the magnitude of change (Δ) in laboratory parameters, clinical index, and Charlson comorbidity index one year after the index date between the two cohorts of patients with HBV-related liver cirrhosis.

	Case (Anti-HBV + Silymarin) (*n* = 319)	Control (Anti-HBV Agent) (*n* = 319)	*p*-Value
Biochemistry, Δ mean ± SD			
ΔCr (mg/dL)	0.08 ± 0.62	0.19 ± 0.71	0.083
ΔNa (mEq/L)	0.70 ± 4.18	−0.05 ± 6.18	0.227
ΔAST (U/L)	−75.66 ± 244.16	−72.95 ± 284.61	0.921
ΔALT (U/L)	−87.2 ± 257.64	−79.81 ± 320.27	0.804
ΔAlbumin (g/dL)	0.48 ± 0.77	0.37 ± 0.87	0.224
ΔHBV-DNA (log10 IU/mL)	−7.31 ± 8.02	−7.44 ± 8.10	0.434
Hemogram			
ΔINR	−0.07 ± 0.30	0.03 ± 0.47	0.038
Clinical index			
ΔMELD score	−1.65 ± 5.22	−0.08 ± 6.75	0.025
ΔALBI score	−0.51 ± 0.79	−0.39 ± 0.92	0.198
ΔCCI (Charlson comorbidity index)	0.77 ± 3.00	−0.30 ± 3.39	<0.0001

Δ: (post–pre) data change one year after the index date; case: silymarin + anti-HBV agents (IFN or nucleoside/nucleotide analogs); control: anti-HBV agents.

**Table 4:** Competing risk analysis for mortality as the primary outcome, with LT ^a^ as the competing risk.

Competing Risk Analysis				
Follow-Up Duration	One Year		Two Years	
Variable	HR (95% CI)	*p*-Value	HR (95% CI)	*p*-Value
Cohort				
Case ^b^	0.43 (0.311–0.61)	<0.001	0.44 (0.33–0.59)	<0.001
Control ^c^	1 (reference)		1 (reference)	

LT ^a^: liver transplantation; case ^b^: silymarin + anti-HBV agents (IFN or nucleoside/nucleotide analogs); control ^c^: anti-HBV agents.

**Table 5:** Competing risk analysis for HCC occurrence as the secondary outcome, with mortality or LT ^a^ as the competing risk.

Competing Risk Analysis				
Follow-Up Duration	One Year		Two Years	
Variable	HR (95% CI)	*p*-Value	HR (95% CI)	*p*-Value
Cohort				
Case ^b^	1.02 (0.71–1.48)	0.907	1.08 (0.78–1.50)	0.651
Control ^c^	1 (reference)		1 (reference)	

LT ^a^: liver transplantation; case ^b^: silymarin + anti-HBV agents (IFN or nucleoside/nucleotide analogs); control ^c^: anti-HBV agents.

## Data Availability

Data and study materials will be made available to other researchers upon request via email, along with their IRB approval document.

## Supplementary Materials

The following supporting information can be downloaded at: https://www.mdpi.com/article/10.3390/ijms25063088/s1.
