# Supplementary material for: Silymarin Synergizes with Antiviral Therapy in Hepatitis B Virus-Related Liver Cirrhosis: A Propensity Score Matching Multi-Institutional Study
Source: Int J Mol Sci. 2024 Mar 7;25(6):3088. doi: 10.3390/ijms25063088 (PMC10970014; doi:10.3390/ijms25063088)
Supplement: Supplementary file 1 [file ijms-25-03088-s001.zip › ijms-2872287-supplementary.pdf]

Supplementary Table S1. Changes in laboratory parameters and clinical indices one year after the index date in the two cohorts of patients with HBV-related liver cirrhosis.

	Case (anti-HBV+ Silymarin) (n=319)			Control (anti-HBV agent) (n=319)		
	Before	After	p-value	Before	After	p-value
Biochemistry, mean±SD						
Cr (mg/dL)	1.17±1.37	1.24±1.4	0.056	1.1±1.5	1.29±1.79	<0.001
Na (mEq/L)	137.62±3.89	138.32±3.84	0.021	137.54±4.26	137.49±5.6	0.929
AST(U/L)	129.72±228.83	54.06±80.44	<0.001	138.3±248.32	65.35±138.87	0.002
ALT(U/L)	125.51±257.16	38.31±30.66	<0.001	136.68±281.5	56.87±159.74	0.002
Bilirubin Total (mg/dL)	3.33±5.09	1.5±1.69	<0.001	3.13±4.31	2.17±3.67	0.033
Albumin (g/dL)	3.34±0.75	3.82±0.7	<0.001	3.27±0.77	3.64±0.79	<0.001
HBV-DNA(log10 IU/mL)	7.37±8.01	6.5±7.17	<0.001	7.47±8.1	6.31±6.68	<0.001
Hemogram						
INR	1.25±0.3	1.18±0.24	0.001	1.2±0.44	1.23±0.45	0.500
Clinical Index						
MELD score	12.68±5.28	11.04±4.77	<0.001	12.44±5.67	12.36±6.47	0.896
ALBI score	-1.84±0.77	-2.35±0.68	<0.001	-1.75±0.8	-2.14±0.8	<0.001
CCI (Charlson Comorbidity Index)	1.93±2.43	2.7±2.86	<0.001	2.11±2.63	1.81±2.58	0.111

Supplementary Table S2

	Case cohort (anti-HBV+ Silymarin) (n=319)		Control cohort (anti-HBV agent) (n=319)		
index year	N	%	N	%	ASMD
2003	0	0	13	4.08	0.658
2004	0	0	6	1.88	
2005	0	0	7	2.19	
2006	23	7.21	14	4.39	
2007	25	7.84	19	5.96	
2008	17	5.33	16	5.02	
2009	18	5.64	12	3.76	
2010	35	10.97	19	5.96	
2011	28	8.78	18	5.64	
2012	16	5.02	21	6.58	
2013	33	10.34	17	5.33	
2014	20	6.27	18	5.64	
2015	25	7.84	20	6.27	
2016	26	8.15	23	7.21	
2017	21	6.58	36	11.29	

2018	19	5.96	19	5.96	
2019	13	4.08	41	12.85	

Absolute Standardized Mean Difference (ASMD) > 0.1 indicating statistical significance

Supplementary Table S3. Competing risk analysis for mortality as the primary outcome, with LT[a] as the competing risk event (adding index year into the model for adjustment)

	Competing risk analysis			
**Follow-up duration**	One-year		Two-years	
**Variable**	HR (95% CI)	p-value	HR (95% CI)	p-value
Cohort				
Case[b]	0.45 (0.31-0.65)	<0.001	0.43 (0.32-0.59)	<0.001
Control[c]	1(Reference)		1(Reference)	

a: LT=liver transplantation; b: Case: Silymarin+ anti-HBV agents (IFN or nucleoside /nucleotide analogs); c: Control: anti-HBV agents.

Supplementary Table S4. Competing risk analysis for HCC occurrence as the secondary outcome, with mortliaty or LT[a] as the competing risk events (adding index year into the model for adjustment)

	**Competing risk analysis**			
**Follow-up duration**	One-year		Two-years	
**Variable**	HR (95% CI)	p-value	HR (95% CI)	p-value
Cohort				
Case[b]	1.06 (0.71-1.59)	0.767	1.01 (0.78-1.59)	0.549
Control[c]	1(Reference)		1(Reference)	

a: LT=liver transplantation; b: Case: Silymarin+ anti-HBV agents (IFN or nucleoside /nucleotide analogs); c: Control: anti-HBV agents.
